# Improving Work-Related Challenges in Psychiatric-Psychosomatic Clinics: Study Protocol for an Internet-Based Needs Assessment and Co-Design of a Training

**DOI:** 10.2196/78047

**Published:** 2025-11-25

**Authors:** Katharina Schiffer, Sonia Lippke

**Affiliations:** 1 Bremen International Graduate School of Social Sciences, Campus Ring 1, Bremen, 28759, Germany, 49 4212004730; 2 Constructor University, Bremen, Germany; 3 Hamburg University of Applied Sciences/Hochschule für Angewandte Wissenschaften Hamburg, Hamburg, Germany

**Keywords:** health care providers, patient safety, occupational well-being, prevention & control, psychology

## Abstract

**Background:**

Medical, psychiatric-psychosomatic facilities are confronted with a variety of daily challenges that affect working conditions, the mental health of employees, and the quality of patient care. This project focuses on the work-related challenges faced by health care professionals in psychiatric-psychosomatic clinics in Germany.

**Objective:**

The aim of the current research is to investigate the interactions between individuals and their social environment, identify psychological and organizational challenges and job demands, and use these findings to inform the development of a participatory, evidence-based intervention.

**Methods:**

This 2-phase research is grounded in the job demands-resources model (JD-R). Study phase (needs assessment) uses a cross-sectional online survey with health care professionals in German psychiatric-psychosomatic clinics to assess job demands, resources, and outcomes in a target sample of *N*=600 participants (power analysis). Study phase 2 (co-design of a training) involves co-creatively designing an intervention based on survey findings through participatory workshops with at least *N*=20 participants. Analyses include regression and moderation tests (SPSS; IBM Corporation) and qualitative data analysis to co-design training.

**Results:**

The recruitment of participants is planned to be finished by December 2025. The co-designing of workshops (phase 2) will be started in February 2026. As this is a study protocol, results are not available yet.

**Conclusion:**

This current research examines the work-related challenges faced by health care professionals in psychiatric-psychosomatic clinics. It is expected that burnout, engagement, and psychological safety will likely emerge as central mediating and moderating variables. As the findings of phase 1 serve as a basis for the development of an intervention, this research seeks to improve the well-being of health care professionals in psychiatric-psychosomatic institutions sustainably.

## Introduction

### Background

Medical, psychiatric-psychosomatic clinics face a multitude of daily challenges, including digital and technological changes [1-4]. With patient safety as the top priority [3-5], health care professionals are under significant pressure, leading to numerous reports of burnout, general job dissatisfaction, and intentions to quit [3]. This exacerbates the existing shortage of specialists in the German health care system [6]. Despite ongoing organizational discussions about systemic changes, the situation for health care professionals is improving only slowly or even stagnating [1,7]. Thus, attention must focus on the individual and team levels to enact meaningful changes.

Due to the unique emotional and psychological demands of treating patients with complex mental health needs, combined with the lack of focus in previous studies, it is essential to understand and support health care professionals better. This is also necessary to ensure high-quality care for patients with mental health disorders and create a work environment where health care professionals feel recognized and cared for [8]. To support all people involved to become and remain empowered by medical internet research to make effective, informed decisions, take control of their health and well-being, and live happier and healthier lives, this research takes up the research gaps and will perform a needs assessment and co-design of a training. The current paper describes the protocol and study design, including its practical and theoretical backgrounds.

### Context and Relevance of the Research

The German health care system, as an example for many others in different countries around the world, is under enormous pressure: health is considered to be one of the highest human goods, but economic constraints, skills shortage, and demographic change are challenging [9]. Accordingly, the health care system is subject to a high level of attention and therefore also high pressure [9]. In Germany, the health care system operates within a tightly regulated economic framework [3], which is incorporated into decisions about changes. In its complexity and its embedding in diverse social contexts (including scientific and technological progress), the health care system is subject to increasingly rapid change [3]. Furthermore, the complexity of the health care system necessarily creates many contradictions in the system, which are: on the team level, between the individual employees and their professional (interests) groups and between the political and corporatist system levels (the level of self-administration and associations [3]).

Authors from various disciplines, like politics, business, public health, and medicine, have already pointed out individual issues and problems in the health care system. It has been shown that changes within and between the levels are possible and necessary [3,10]. These problems, requirements, and perspectives relate to both inpatient and outpatient care within the German health care system.

Hospitals are a central pillar of the health care system. The complexity of the health care system is represented in a concentrated (perhaps even potentiated) form in them: staff from a wide variety of disciplines with different levels of seniority and training have to provide round-the-clock (and often emergency) care for a wide variety of patients and work together to this end [11]. The complexity and resulting problems of the health care system turn hospitals into a so-called “high-risk organizations” [3]. Dorn [12] views the hospital as a complex organization in which the different functional, organizational, and professional logics collide. The resulting interrelationships produce organizational conflicts and uncertainties.

Psychiatric-psychosomatic clinics are an integral part of this health care system. The collective term covers clinics that treat patients with psychiatric diagnoses. Some clinics specialize in certain clinical conditions, others in a certain clientele or catchment area (due to care mandates from the federal states). They vary in size (number of patients, employees, beds, and wards) [8]. Clinics are usually structured similarly to hospitals that primarily treat physical illnesses and are also grouped together with them in scientific studies [10]. The problems appear somewhat similar: the care index is too low due to staff shortages and financial reasons or reasons at the organizational level and employee satisfaction is reduced [1].

There are some crucial and aggravating factors in psychiatric-psychosomatic clinics that distinguish them from other inpatient facilities that specialize more in physical health problems: first, the relative recency of the branch. This is due to a prolonged stigmatization that has persisted over time and continues to exist in a weaker form. The product is the stigmatization of mental illness and the underplaying of mental illness. Second, the type of diagnoses that are treated there: the treatment of mental disorders is different from that of physical disorders. In particular, it is more difficult to determine whether a previous treatment had negative side effects or whether it has been applied successfully and correctly [10]. It is only possible to determine relatively roughly whether a specific procedure was wrong or inappropriate in a specific situation with a specific patient. The treatment guidelines for certain clinical pictures or a certain therapy school-specific procedure leave a relatively large amount of degree of freedom [8].

The work in psychiatric-psychosomatic clinics is organized in a team of different professions in which different functions, organizational, and professional logics collide [12]. These interdisciplinary conditions of multiprofessional teams [12] create challenges like communication problems between the different professional groups, role ambiguity, as well as hierarchical differences [12].

Health care professionals in psychiatric-psychosomatic institutions are exposed to numerous risks and challenges [13,14], which lead to health problems and illnesses, such as burnout [15-17]. As a consequence, there is frequent staff turnover and high fluctuation between wards [10]. Job satisfaction may also be reduced [1]. Psychiatric-psychosomatic facilities face a variety of daily challenges that impact working conditions, employee mental health, and the quality of patient care. With patient safety as a top priority, health care professionals are under considerable pressure, often leading to burnout, job dissatisfaction, and the intention to change careers. This is exacerbating the already existing workforce shortage in the German health care system. Despite ongoing discussions about social systems, ranging in complexity and dynamically changing over time, and systemic changes, the situation for health care professionals is only slowly improving. There are unique problems in psychiatric care: distinct structures and processes in psychiatric settings complicate the direct application of solutions from other health care domains.

### Collaborative, Co-Creative Approaches in Times of Internet-Based Medical Services

Research into the collaborative aspects of digital health interventions could potentially address various challenges within the health care system and related services in an increasingly digitalized world. Specifically, investigating effective strategies for including researchers, health care professionals, developers, and target groups (such as individuals with disabilities, patients, or clients considered as co-creators) may help reduce costs, enhance user responsibility and engagement, and improve the likelihood of successful implementation and sustainability in practice. This is particularly relevant when developing, marketing, and implementing new technologies [4]. Collaborative approaches in research and practice have been used over the past century [18].

The dissolution of barriers between researchers and target groups can be traced back to Lewin’s use of Action Research in 1946 [3,6,10,19,20]. In Action Research, the researcher is also a member of the target group and takes part in evaluating an intervention (or an action, as termed by Lewin). Both researchers and target groups, such as health care professionals, engage in a process of reflection to test new actions; planning how to test the new action, acting, and finding results about the action.

Another example of the partnership of researchers and target groups is Kristen Nygaard’s work in 1972 in Norway [21]. Nygard was a computer scientist tasked with developing training material on workplace information technology (IT) systems. To develop the material, Nygaard recruited 4 local union shops geographically spread over southern Norway. With his team, Nygaard developed a textbook on “Data processing, planning and control,” which allowed employees to leverage IT systems; Nygaard’s work is regarded as the precursor to participatory approaches [18].

Different terminologies and definitions for collaborative (research) practices also arose, relating to technology and digitalization, such as the above-described co-creation, co-design, and participatory approaches. A reliable management of collaboration is needed, aiming for improving social equality and participation by using technology [4]. The involvement of health care professionals in psychiatric-psychosomatic institutions requires recognizing them as valuable individuals with unique needs, in addition to generic requirements. Their involvement and responsibilities require potential bridges, such as supporting individuals to “translate” for them or technology to aid communication and interaction. As digital demands are faced in the workplace, their efficient use could help this population in times of staff shortage, increased diversity, and workplace requirements [18].

Internet-based, technological, and digital solutions range from electronic patient-reported outcome measures to eHealth interventions, including online interventions and apps empowering individuals with topics like healthy lifestyles or safe communication and shared decision making [22,23], facilitating group psychotherapy from home [24,25], or monitoring biomedical markers and getting biofeedback [26-28]. Digital solutions also involve technological support systems, like digital psychotherapy for mental health disorders, robot systems for individuals with dementia, and exoskeletons to help people who lost their legs walk again. While there are many advantages, adoption might be burdensome, or their use might be hampered. Thus, health care professionals in psychiatric-psychosomatic institutions need to know about how to use these options and prevent related problems; otherwise, they do not understand their potential, do not start with it, or may drop out quickly. Thus, co-creation and co-design can make a significant difference with its actual impact, including effectiveness [18].

### Previous Evidence

Due to the unique emotional and psychological stresses associated with treating patients with complex mental illnesses, it is crucial to examine the working conditions of health care professionals in this field in more detail. Previous studies [29,30] have addressed specific challenges in psychiatric-psychosomatic clinics, but these have often been limited in scope or focus. While the workloads of nursing staff are generally considered to be relatively well documented [29,30], little is known about nursing staff in inpatient psychiatric health and nursing care [31].

Another challenge for research is that social complexity within the health care system, including the use of technology adoptions, leads to contradictions, organizational tensions, and uncertainty [12]. Despite this, there is currently a lack of research that examines the team level—the space between individual employees, their professional groups (nurses, psychologists, and physicians), and the broader political and organizational systems [3]. Yet, clinical work is typically structured around teams, which brings specific challenges for interprofessional collaboration and coordination among staff. Due to the great pressure and risk to job satisfaction and psychological safety, concepts such as “just culture” have been introduced to foster a supportive work environment [3].

A systematic overview of the current literature shows that various strategies and approaches to psychological safety exist [3]. Above all, the responsibility of leadership is discussed [3]. At this stage, for example, the case is being made for a safety culture for the entire system, including all individuals, professional groups, teams, organizations, associations, and the health care system [10]. Previous research has identified the challenges with technology and opportunities for quality improvement, research, and policy development [4]. Despite these opportunities to support health care professionals in understanding their performance, engaging in reflective practice, and enhancing professional and workplace learning, there is only limited systematic research [4]. At this stage, approaches are being pursued that no longer focus solely on patient safety but concentrate more on a patient- and employee-oriented risk and safety culture. Psychological safety is fundamental to all these aspects [3,32].

Although promising approaches exist, few have been implemented concretely in the specific context of psychiatric-psychosomatic clinics, highlighting the need for more systematic and theory-based research [33,34]. Above all, the team composition with the special role of psychologists and the change in social dynamics, as well as their role itself, has to be further researched [35]. In order to find out how health care professionals deal with these conditions, difficult situations, or new challenges in clinical practice, Bandura [36] formulated in his social-cognitive theory that self-efficacy beliefs have a greater influence on motivation, emotions, and actions than objective factors such as ability level [36-39]. Self-efficacy is a key factor in human behavior that influences performance, motivation, and resilience [40,41]. Heinrich et al [42] showed in their studies on the health care sector that high self-efficacy is a protective factor against burnout [42,43]. In the context of health psychology, self-efficacy plays a prominent role: higher self-efficacy is associated with better health (outcomes) [8,44]. Self-efficacy has been successfully integrated into interventions [45], though applications in psychiatric-psychosomatic contexts remain rare or underexplored. Most interventions primarily target individuals rather than teams, despite the central importance of team dynamics in this context [46].

The job demands-resources (JD-R) model [47,48] provides a suitable framework for analyzing the working conditions of health care professionals in psychiatric-psychosomatic clinics. Key indicators, such as high sickness rates [15,16], frequent staff turnover and interward fluctuations [49], technologization [4], and reduced job satisfaction [1], highlight the relevance of this model in capturing occupational stressors and resource deficits.

### Theoretical Framework: the JD-R Model

**Figure 1.**
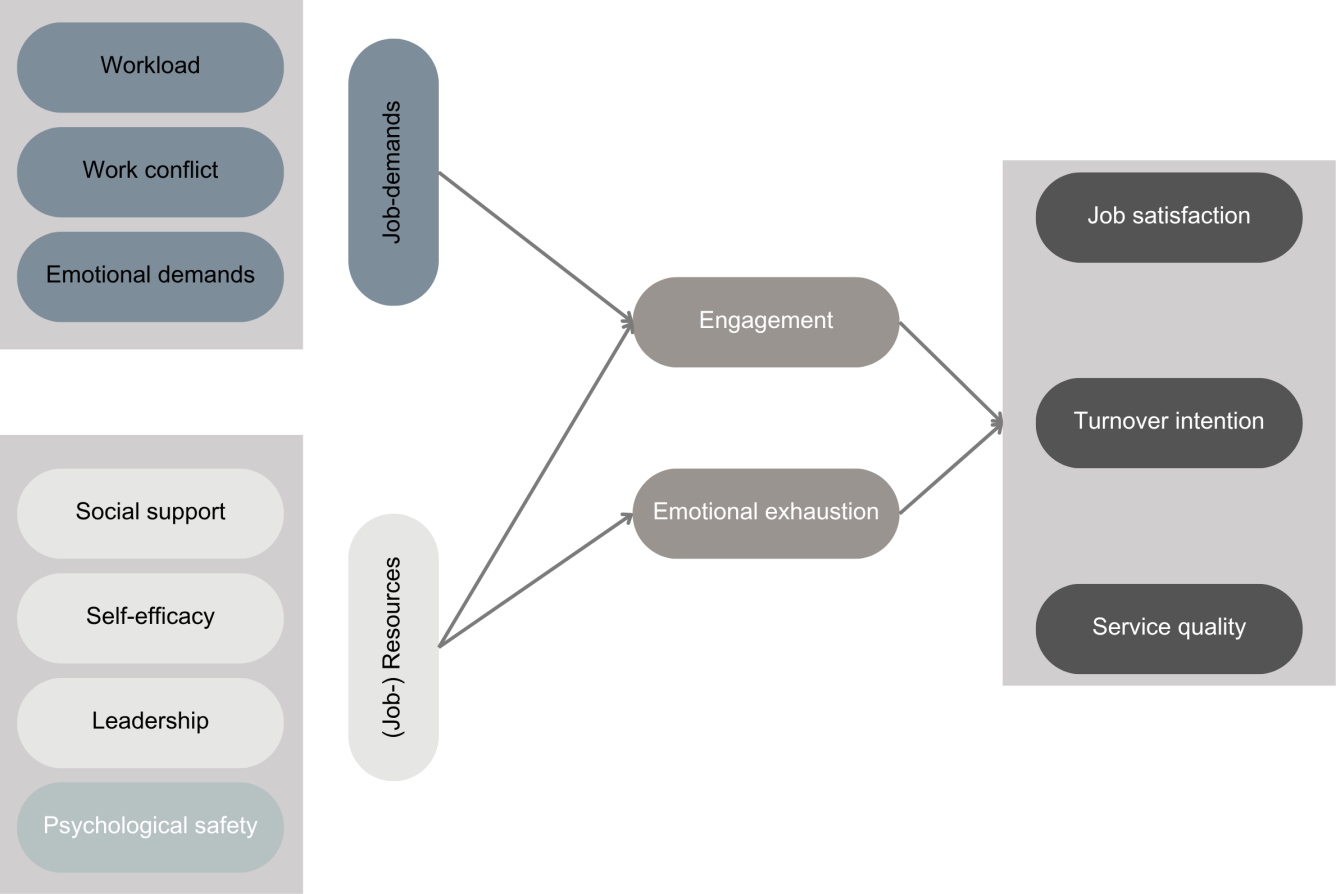
The job demands-resources model.

The JD-R model [47] (Figure 1) emphasizes the importance of job demands (high workload and emotional stress) and job resources (social support and self-efficacy) for the job satisfaction, engagement, and burnout risk of employees.

The JD-R theory explains how job demands and job resources influence job performance through employee well-being (including burnout and emotional exhaustion and work engagement) and how employees use proactive as well as reactive work behaviors to impact job demands and resources [50,51]. However, the following should be noted for the psychiatric-psychosomatic health care sector: the different structures and processes in psychiatric settings described above make it difficult to directly apply solutions from other health care sectors.

Against the background of the working conditions in psychiatric-psychosomatic clinics described above, it is precisely the 2 fundamental concepts of the model—the concept of burnout and the concept of job demands—that make the JD-R model attractive for research into this area of health [52]. The model integrates various job stress and motivational perspectives [50,51].

Bakker et al [47] underpin the theoretical and empirical conceptualization of the constructs of burnout and work engagement with numerous (meta) studies on situational factors and individual factors of burnout and work engagement. The JD-R model is a widely used instrument and has been applied in numerous studies [53]. However, the distinct structures and processes in psychiatric settings described above complicate the direct application of solutions from other health care domains.

The findings of Halbesleben [54] are relevant to the present research, as he proved that job resources and self-efficacy go hand in hand with work engagement. Mäkikangas et al [55] also add self-efficacy, optimism, and self-esteem. With regard to health-related outcomes, Bakker et al [47] explained that there is wide empirical evidence showing that the experience of burnout leads to several health-related issues. Yang and Hayes [49] conducted a systematic review of 44 studies carried out between 2009 and 2020. They concluded that psychotherapists enduring burnout are at risk of developing a wide range of health-related problems, including anxiety and depressive disorders, secondary traumatic stress, back and neck pain, sleep deprivation, and insomnia [47]. In a systematic review, Patel et al [56] showed that physicians’ burnout leads to serious outcomes, such as increased risk of malpractice or increased number of medical errors.

Accordingly, this research examines how these factors influence job satisfaction, the intention to change jobs, and subjective service quality. A particular focus is placed on the mediators of burnout and commitment, as well as on the role of psychological safety as a moderator between job demands and the outcomes mentioned [57]. Figure 1 shows the structure of the relationships in the model. A special focus is placed on the mediator’s burnout and emotional exhaustion and engagement, as well as on the role of psychological safety as a moderator between job demands and the outcomes mentioned (job satisfaction, the intention to change jobs, and subjective service quality). By investigating these relationships, a deeper understanding of the psychological factors that influence health care professionals in psychiatric-psychosomatic clinics can be gained.

For the current research, job demands include the following: workload, work conflict, and emotional demands. Further, the job resources include the following factors: social support, self-efficacy, leadership, and psychological safety. The selection is justified as follows: 9 central propositions are developed in the JD-R theory, which will guide the outlined research [47].

The first proposition on the basis of the JD-R theory is that all job characteristics can be modeled using 2 distinctive categories, namely job demands and job resources [58]. Job demands are defined as the physical, psychological, social, or organizational aspects of the job that require sustained physical, cognitive, or emotional effort and are therefore associated with certain physiological or psychological costs [58]. In contrast, job resources are defined as the physical, psychological, social, or organizational aspects of the job that have motivating potential, that are functional in achieving work goals, that regulate the impact of job demands, and that stimulate learning and personal growth [51].

The second proposition of JD-R theory is that job demands and resources instigate 2 different processes: in the first process, the health impairment process, the frequency and severity of job demands leads to increased effort and may lead to job strain, exhaustion, and health problems [58]. A study on health care professionals, Molero et al [59] shows that job demands (eg, time pressure and emotional stress) lead to burnout, which in turn acts as a mediator between demands and negative outcomes (eg, job satisfaction and intention to leave). In the second process, the motivational process, job resources (eg, skill variety, social support, and feedback) satisfy basic psychological needs and produce work engagement. The experience of work engagement consequently leads to creative performance [60]. In a multinational study in 4 organizations, Schaufeli and Bakker [61] were able to show that work resources promote commitment, which in turn refers to the mediating role of commitment.

The third proposition is that job demands and resources have a multiplicative impact on employee well-being (including burnout and work engagement). Several studies have shown that job resources such as skill variety, performance feedback, and opportunities for recovery can mitigate the impact of various job demands (eg, workload, cognitive demands, and emotional demands) on strain, including psychological distress, burnout, and psychosomatic complaints [47,62-64]. Personal resources are positive self-evaluations that refer to individuals’ sense of their ability to control and impact their environment successfully [65]. Thus, the fourth proposition on the basis of the JD-R theory is that personal resources, such as optimism, self-efficacy, and resilience, have a reciprocal relationship with job resources. This means that employees with more personal resources are expected to also have access to more job resources, and vice versa. Indeed, using a longitudinal design, Xanthopoulou et al [66] found that employees who were more self-efficacious and optimistic also reported higher levels of autonomy, performance feedback, and opportunities for growth over time. In addition, job resources have had a lagged positive effect on personal resources. Schaufeli and Bakker [61] and Bakker et al [62] show that high job resources not only increase engagement, but also job satisfaction. A meta-analysis by Podsakoff et al [67] confirms that work resources such as social support and feedback reduce the intention to change, while high demands increase it.

A further proposition (proposition 9 according to Bakker et al [47]) of the model that is fundamental to this research is that of the loss cycle, which states that job strain is both an outcome and a predictor of dysfunctional and job demands. Thus, over the course of time, employees may enter a loss cycle. Specifically, when employees experience job strain in the form of exhaustion, anxiety, or depressive complaints, they deplete their energy resources and engage in dysfunctional coping.

In this research, the factor of psychological safety is added to the job resources. It plays a central role in the context of psychiatric-psychosomatic care. Psychological safety has been studied less frequently in this context to date and is also not explicitly listed as a job resource by Bakker et al [47,50]. However, others also include psychological safety as a job resource and thus justify psychological safety climate as a precursor to conducive work environments [57]. Psychological safety in the health care workplace—as mentioned before—is fundamental. It can also be seen as a mediator for job satisfaction [3].

Psychological safety is therefore a central element of this research. With regard to psychological safety, Hecker [3] emphasizes that the current discussion is moving away from the following “one-sided” definition of patient safety [68]. The current debate is about a so-called “patient- and employee-oriented risk and safety culture” [3]. A working environment in which patients and employees feel accepted and respected is characteristic of and fundamental to psychological safety [3,32,69]. In order to support employees, it is necessary to create a psychologically safe environment [3].

Woodward speaks of a “restorative just culture,” where nobody is afraid to speak up and where employees are supported and encouraged without blame, especially when mistakes happen [3]. Although the just culture is well founded [3], everyday life in hospitals is mostly quite different: mistakes are tried to be concealed and employees avoid speaking up out of fear of negative consequences. There are hardly any publications on how to implement the just culture and thus also the psychological safety of employees [3].

By examining these relationships between job demands and job resources in relation to job satisfaction, turnover intention, and service quality (see Figure 1), a systematic understanding of the psychological factors that influence health care professionals in psychiatric-psychosomatic clinics can be used for designing innovative interventions.

### Research Objectives and Questions

The aim of this paper is to investigate the interactions between individuals and their social environment in health care professionals working in psychiatric-psychosomatic clinical settings in Germany. The ultimate goal is to co-design a tailored intervention to improve their well-being.

Grounded in the JD-R model [47], the research adopts a 2-phase design with the overarching research goal of identifying psychological and organizational challenges and using these findings to inform the development of a participatory, evidence-based intervention. Thereby, we build on previous evidence and developments (for instance [4]).

Phase 1 serves as a comprehensive needs assessment. It aims to investigate how the assessed resources and demands of health care professionals impact job satisfaction, turnover intention, and subjective service quality. In this context, the research particularly considers how emotional exhaustion and engagement function as mediators, and how psychological safety may act as a moderator in these associations. The results of phase 1 will form the empirical foundation for a context-sensitive intervention tailored to the specific needs of clinical staff to be developed in phase 2.

The central research question for phase 1 is: How do the assessed resources and demands interrelate with job satisfaction, the intention to leave the job, and the subjective service quality of health care professionals in psychiatric-psychosomatic clinics? To address this question in greater depth, phase 1 focuses on the following specific subquestions: how do the surveyed resources and demands of health care professionals impact job satisfaction, turnover intention, and subjective service quality? What roles do burnout, engagement, and psychological safety play as mediators and moderators in these relationships? What are the key challenges and resources identified by health care professionals in their daily work within psychiatric-psychosomatic settings?

Drawing on the theoretical assumptions of the JD-R model, the research tests the following hypotheses: (Hypothesis 1) higher job demands are negatively associated with job satisfaction and service quality and positively associated with turnover intention. (Hypothesis 2) Higher job resources are positively associated with job satisfaction and service quality. (Hypothesis 3) Burnout acts as a mediator between demands and outcomes. (Hypothesis 4) Engagement mediates the positive effect of resources on the outcomes. (Hypothesis 5) Psychological safety moderates the relationship between demands and negative outcomes.

Building on the findings of the first phase, phase 2 pursues the primary objective of co-creatively developing an intervention aimed at enhancing the well-being of health care professionals in psychiatric-psychosomatic settings in times of digitalization and increased impact of technology. This participatory development process involves key stakeholders and practitioners to ensure practical relevance, acceptability, and contextual sensitivity. In addition to the intervention design, phase 2 also aims to evaluate the feasibility and acceptability of the intervention and to explore its preliminary potential to improve job satisfaction, reduce turnover intention, and enhance perceived service quality.

Taken together, this 2-phase research project seeks first to systematically examine the demands and resources present in psychiatric-psychosomatic practice and their impact on central psychological and occupational outcomes, and second, to design an evidence-based intervention that incorporates the perspectives of those directly affected by these challenges, especially in times of increased technological demands.

## Methods

### Overview

This research uses a 2-phase approach to investigate the interactions between individuals and their social environment to study the self’s embeddedness in diverse social systems. Concretely, the work evaluates work-related challenges among health care professionals in German psychiatric-psychosomatic clinics and develops a tailored intervention. Phase 1 consists of a cross-sectional online survey to identify key stressors and resources, while phase 2 involves a participatory co-creation process to design an evidence-based intervention [70].

### Phase 1: Internet-Based Needs Assessment

#### Study Design

A cross-sectional online survey will be conducted to examine associations between job demands, job resources, and work-related outcomes (job satisfaction, turnover intention, and perceived service quality) using the JD-R model [47,48]. Data will be collected at a single time point via the SoSci Survey [71] platform (SoSci Survey GmbH), ensuring efficient and scalable data collection.

#### Target Group and Recruitment

We conducted a priori power analysis using G*Power 3.1 [72] to determine the required sample size for our primary analysis incorporating 7 predictors (3 job demands and 4 job resources). The analysis indicated that n=178 participants would be needed to detect small-to-medium effects (f²=0.15) with 80% power at *α*=.05. This effect size estimate is empirically supported by: (1) meta-analytic findings in JD-R research reporting comparable effects (f²≈0.12‐0.18 [73]), (2) health care studies of psychological safety (*β*=.32‐.41 [32,52]), and (3) recent JD-R meta-analyses in mental health settings (f²=0.10‐0.20 [52,74,75]).

For subgroup analyses across 3 professional groups (nurses, psychologists, and physicians), we calculated that 159 participants per group (n=477 total) would be required to detect medium-sized effects (η²=0.06) with 80% power [76,77]. Accounting for 20% anticipated attrition, we established a final target of n=600 (200 per group). This sample size follows recommendations from occupational health research [51,53] and provides 70% power for exploratory analyses of experience levels (d=0.65 [74]), while maintaining practical feasibility in clinical research settings.

Eligibility criteria include: (1) age ≥18 years, (2) current employment in a psychiatric-psychosomatic clinic, and (3) sufficient German language proficiency. Exclusion criteria apply to nontarget professions (eg, administrative staff) and professionals outside specified clinical settings. Primary recruitment will occur through clinic administrators who will distribute survey invitations via email. Emails will be sent out via a list of clinics that primarily treat patients with psychological/psychosomatic disorders, or that have specific wards that focus on these patients. Recruitment will be limited to Germany. Supplementary methods include targeted social media advertisements and informational posters in participating institutions. Participation is voluntary, with guaranteed anonymity and the right to withdraw without consequences.

### Data Collection

Data collection for this study will be conducted through an online survey to be administered via SoSci Survey [71], with an estimated completion time of 8 minutes. The survey will use standardized measures adapted from established instruments to assess key constructs within the JD-R [51], with primary reliance on the Copenhagen Psychosocial Questionnaire (COPSOQ [78]).

Job demands will be assessed through three key dimensions. Workload will be measured using five items evaluating work pace and time pressure (eg, “How often do you have to work very fast?”), to be rated on a 5-point frequency scale (1=always to 5=never), adapted from the COPSOQ [78]. Work conflict will be assessed through 4 items examining interpersonal tensions and workflow disruptions (eg, “Do you often have arguments with some colleagues?”) using the COPSOQ’s conflict scale (4-point agreement scale; 1=not at all to 4=completely true). Emotional demands will be evaluated through items assessing emotional strain (eg, “Is your work emotionally demanding?”) to be rated on 5-point intensity and frequency scales from the COPSOQ [78].

Job resources will be measured through several dimensions. Social support will be assessed using 4 items evaluating collegial and supervisory support (eg, “How often do you receive help from colleagues when needed?”), to be rated on a 5-point frequency scale (1=always to 5=never) from the COPSOQ [78]. Self-efficacy will be measured through nine items assessing problem-solving confidence and adaptability (eg, “I can always find solutions to difficult problems”), using a 4-point agreement scale (1=not at all to 4=completely true), based on established self-efficacy scales [79]. Burnout refers to a work-related state of exhaustion and a sense of cynicism. In contrast, work engagement is a positive motivational state of vigor, dedication, and absorption [79].

Leadership quality will be evaluated through 4 items examining motivational and fair leadership behaviors, to be rated on a 4-point agreement scale adapted from the COPSOQ. Psychological safety will be assessed using seven items (eg, “In this team, difficult topics can be discussed openly”) adapted from Edmondson’s [69] team psychological safety measure, to be rated on a 7-point Likert scale (1=strongly disagree to 7=strongly agree).

Psychological states will include work engagement and emotional exhaustion. Work engagement will be measured using three items from the Utrecht Work Engagement Scale (UWES [80]) assessing energy, enthusiasm, and absorption (5-point frequency scale). Emotional exhaustion will be evaluated through 3 items (eg, “How often are you emotionally exhausted?”) to be rated on a 5-point frequency scale from the COPSOQ [78].

Outcome variables will comprise job satisfaction, turnover intention, and service quality, all adapted from the COPSOQ [78]. Job satisfaction will be assessed through seven dimensions (eg, satisfaction with colleagues, management, and salary) using 5-point Likert scales. Turnover intention will be measured with two items assessing frequency of thoughts about leaving the profession or job (5-point frequency scale). Service quality will be evaluated through 3 items (eg, “The care provided on my unit is reliable”) using a 7-point Likert scale.

Demographic and occupational characteristics will include comprehensive background information to enable subgroup analyses: Age (continuous variable in years), gender (male, female, diverse, and prefer not to say), professional role (nurse, psychologist, and physicians), years of professional experience (continuous), weekly working hours (continuous), shift work status (yes, no), team size (number of colleagues typically worked with), and participation in continuing education (hours per year).

All measures have been selected based on their established reliability and validity in prior research. The survey will maintain participant anonymity and comply with GDPR requirements. Electronic consent will be obtained before participation, and participants will be able to withdraw at any time without penalty. The standardized administration through SoSci Survey will ensure consistent data collection across participants.

### Statistical Analysis

The statistical analysis will be conducted using SPSS Version 29 (IBM Corp). Initial data screening will include comprehensive checks of distributional properties, focusing on skewness (absolute values <2) and kurtosis (absolute values <7), along with identification of multivariate outliers using Mahalanobis distance (*P*<.001). Assumptions for parametric tests will be verified through examination of multicollinearity (variance inflation factors <3) and homoscedasticity using the Breusch-Pagan test.

Descriptive statistics including means, SDs, and frequencies will be computed with 95% CIs to characterize the sample. Bivariate relationships will be assessed using Pearson correlations, with false discovery rate correction applied to control for multiple comparisons. Hierarchical regression analysis will be used to examine predictors of primary outcomes, with change in *R*² reported at each step to evaluate the contribution of different predictor blocks. For group comparisons between professions, one-way ANOVAs will be conducted with Games-Howell post hoc tests to account for potential unequal variances, accompanied by effect size estimates (η²) with 90% CIs.

Missing data will be handled through a systematic approach beginning with an examination of missing patterns using Little’s MCAR test. Where data are missing at random, multiple imputations with 50 iterations will be performed using predictive mean matching. Sensitivity analyses comparing results from complete cases versus imputed datasets will be conducted to evaluate the robustness of findings. All analyses will use 2-tailed tests with an alpha level of .05, and effect sizes will be reported in accordance with APA Style Journal Article Reporting Standards [81].

### Phase 2: Co-Design of Training for Medical Staff

#### Development of an Intervention

Building on findings from phase 1, we will use an adaptive participatory action research framework to co-create tailored interventions [4,70]. Co-design means designing an intervention together with the target group to make interventions ideally fitting and engaging, which lead to optimal outcomes. The intervention can be improved in terms of service quality and more satisfactory services for all individuals, including ones with disabilities [82].

Collaboration between different groups of stakeholders to achieve mutually beneficial outcomes is perceived as more effective, trustworthy, and sustainable [83]. The terms co-design and co-creation encompass collaborative approaches characterized by collective creativity, involving diverse views of relevant stakeholders in developmental and problem-solving processes aimed at achieving a shared goal [84]. Participatory health research considers the challenges and advantages of collaborative approaches between individuals with lived experience and organizations.

Participants are seen as active partners, equally important and with equal expertise as health care providers or researchers [18]. This can be ensured by considering all individuals as co-facilitators or stakeholders and active participants in the design of service and care, including accreditation processes. While participation remains more with consultation (like asking about needs, options, or preferences), co-approaches relate more to

- partnership
- shared decision making,
- shared power
- accountability and responsibility
- active control given to all stakeholders.

Benefits of such actions can improve health outcomes and satisfaction [82]. Thus, co-creation, co-design, and co-production harbor great potential for use in different research disciplines due to the active involvement of relevant stakeholders providing their perspectives in the context of intervention design, implementation, and evaluation. Participatory and co-approaches can improve research processes as well as scientific outcomes, including dissemination potential [82,83].

The concrete procedure will be determined through ongoing collaboration with stakeholders, allowing the methodology to evolve based on initial results and practical constraints identified in phase 1. Rather than predetermining group compositions or session numbers, we will adopt a responsive approach where workshop structures emerge organically from the needs assessment data with at least 20 participants. The same holds true regarding the analysis of transcribed verbal data: qualitative content analysis will use software like MaxQDA (VERBI Software GmbH), QCAmap (Association for Supporting Qualitative Research), or QualCoder (Dr. Colin Curtain).

The development process will follow established principles of co-creation in health care settings [70]. Dynamic facilitation techniques will be used to identify intervention priorities while respecting profession-specific perspectives [70,82]. Subsequent refinement cycles may involve either homogeneous or heterogeneous groupings, depending on the emerging themes [70]. For instance, separate sessions might be convened to address profession-specific challenges identified in phase 1, while interdisciplinary workshops would focus on systemic solutions. The development of this participative training for medical staff offers all team members the opportunity to contribute regardless of hierarchy, experience, or personality. This is in line with Edmondson’s call for an inclusive team culture, which contributes to psychological safety and ultimately leads to an innovative corporate culture as proposed by Edmondson [85].

#### Expected Components and Feasibility

The intervention design will be grounded on outcomes of phase 1 and the JD-R framework (Figure 1), directly addressing the specific demands and resources identified as most salient in phase 1 [86]. Drawing on the quantitative and qualitative findings from the initial study phase, the intervention will target modifiable factors that emerged as critical predictors of well-being, while remaining acutely sensitive to the substantial time constraints and emotional burdens characteristic of psychiatric-psychosomatic care settings [87].

Core components will likely focus on three interrelated domains informed by the JD-R analysis. First, stress management elements will address the most prevalent job demands identified, potentially including modules on emotion regulation techniques tailored to high-intensity clinical encounters and strategies for managing workload pressures [88]. These will be designed as brief, skill-based trainings that can be readily integrated into daily practice [89]. Second, team communication enhancements will aim to bolster psychological safety and social support, resources that may emerge from phase 1 as particularly impactful buffers against strain [90]. This could involve structured protocols for interdisciplinary case discussions or conflict resolution processes adapted to mental health care contexts. Third, resource-building components will target both organizational and personal protective factors, such as increasing job control through workflow adjustments or strengthening recovery experiences during nonwork time [91].

The format and delivery methods will be carefully developed through participatory design to minimize additional burden on this already overextended workforce [92]. Early stakeholder input suggests hybrid approaches combining digital and in-person elements may offer an optimal balance between effectiveness and feasibility [93].

Potentially, brief digital micro-interventions delivered through mobile platforms could provide just-in-time support during workdays, while selective in-person sessions would focus on team-based skill building. Particular attention will be given to ensuring all components can be feasibly implemented within existing workflows, potentially through strategies like manager-protected time for participation or integration into regular team meetings [94].

Implementation planning will incorporate systematic assessment of time requirements and workflow impacts, with iterative refinements to reduce participation barriers [95]. The intervention will be designed not as an added obligation, but as a supportive framework that actively reduces current strains while building sustainable resources, aligning with the JD-R perspective’s dual focus on demand reduction and resource enhancement [96]. This approach recognizes that for overburdened health care professionals, even well-intentioned interventions can become counterproductive if not thoughtfully adapted to real-world constraints [97].

### Ethical Considerations

The study 1 was conducted in full compliance with the European Union’s General Data Protection Regulation (GDPR; European Parliament, 2016, Art. 6(1)(a)) and received ethical approval from the Ethics Committee of Bremen International Graduate School of Social Sciences (BIGSSS), Germany (No. BIGSSS20241127; approved on November 27, 2024). A comprehensive data protection plan was approved on the 11.03.2025 by the responsible data security officer during the study design process. Participants receive detailed written information about the study’s purpose, procedures, and data handling practices before providing electronic informed consent through the SoSci Survey platform. Participation is voluntary, and participants can withdraw with no consequences at any time. They did not receive any material or financial incentive for their participation.

Data collection followed strict anonymization protocols, with no collection of personally identifiable information (eg, names, IP addresses, or workplace identifiers). The approved data protection plan specified a 10-year retention period for research data, after which secure deletion would occur following institutional protocols. The plan included comprehensive access logging and procedures for potential data breaches compliant with GDPR requirements. Participants were informed of their rights under GDPR, including access, correction, withdrawal, and complaint procedures. The authors can be contacted by the participants for any complaints or concerns via a provided email address in the survey.

Phase 2 will follow established ethical protocols in line with institutional and GDPR requirements. Before beginning, we obtained the necessary ethical approval for all activities, including specific authorization for the qualitative components. Participants are fully informed about the study and provide consent before taking part.

Confidentiality will be prioritized throughout the qualitative data collection. Workshop and interview recordings will be transcribed with all identifying details removed. The research team will store and handle all data securely using encrypted systems with controlled access.

The approach is designed to protect participants while allowing for necessary adjustments during the intervention development process. Regular reviews will help ensure ongoing compliance with data protection standards.

## Results

The data collection for phase 1 began in March 2025 and is scheduled to be closed by December 2025. Data analysis will follow, and phase 1 is scheduled to be finished by January 2026. Ethics approval has been obtained.

Phase 2 will be built on that. The co-creative workshops are planned for March and April 2026. Phase 2 is scheduled to be finished by May 2026.

No results are available at this stage. This paper reports only the study protocol.

## Discussion

### Anticipated Outcomes and Significance

This study aims to research work-related challenges faced by health care professionals in psychiatric-psychosomatic clinics. It identifies job demands (eg, workload, work conflicts, and emotional demands) and work resources (eg, social support, self-efficacy, leadership qualities, and psychological safety). Based on these findings, this study aims to fill the gaps in research on the working conditions of health care professionals and to develop evidence-based interventions that improve staff well-being and the quality of patient care [32,51-53].

The first phase of the study described above is intended to identify the most important professional requirements and resources that are relevant for mental health professionals in psychiatric-psychosomatic clinics. Hypotheses we anticipated on burnout, engagement, and psychological safety will be tested. In doing so, key hypotheses derived from the theoretical framework are tested, specifically, (Hypothesis 1) the negative association between job demands and job satisfaction and service quality, (Hypothesis 2) the positive association of job resources with these outcomes, (Hypothesis 3) the mediating role of burnout, (Hypothesis 4) the mediating role of engagement, and (Hypothesis 5) the moderating role of psychological safety.

To our knowledge, there are currently few published, comparable studies on daily practice in psychiatric-psychosomatic clinics [14,29,31]. The findings will provide a detailed picture of professional challenges and support structures (in line with the primary research question and hypotheses [Hypothesis 1–Hypothesis 5]). The second phase of the research will result in a co-designed intervention tailored to the unique needs of the target group [70]. This 2-phase approach bridges assessment and action, generating both empirical insight and practical solutions.

The strengths of this 2-phase approach lie on the one hand in the use of the well-established theoretical JD-R model [47]. It offers a broad sample including multiple health care professions. On the other hand, there is an advantage in the combination of quantitative (survey) and qualitative (intervention design) methodologies. The survey records individual perceptions and assessments of job requirements, resources, job satisfaction, intention to change jobs, and service quality. By describing these relationships, a deeper understanding of the psychological factors that influence health care professionals in psychiatric-psychosomatic clinics will be gained [4]. These participatory methods ensure high contextual relevance and potential acceptance. Another strength of this research is the high ethical and data protection standards observed throughout.

There are some potential limitations and suggestions for follow-up of this research that should be noted: The cross-sectional design of phase 1 does not allow for causal conclusions. This would be valuable in order to represent social dynamics within a team of health care professionals. While a longitudinal approach would be ideal, in these 2 phases, it was not feasible due to practical constraints, including the need to reduce participant burden, anticipated staff turnover, and limited comparability across time points. There is also a risk of self-report bias due to the use of only subjective survey responses. To mitigate this, we used neutral question phrasing and ensured anonymity. There is furthermore a risk of potential sample bias due to the self-selection of participants. To address this, recruitment materials emphasized confidentiality, and targeted outreach was conducted to encourage participation across diverse facilities.

Future research should aim to validate these findings with nonreactive data, in international contexts, and across other health care disciplines. Longitudinal studies are needed to explore changes over time in job satisfaction, turnover intentions, and health care quality. Furthermore, RCTs are recommended to evaluate the effectiveness of the co-designed intervention. Follow-up studies should also assess long-term outcomes, including staff turnover rates and patient-related service quality metrics. This research provides a foundation for such future investigations and is planned to be published as two papers (phase 1 and 2) in scientific journals.

This study significantly contributes to answering the central research question of how the resources and requirements influence job satisfaction, the intention to change jobs, and the subjective service quality of health care professionals in psychiatric-psychosomatic clinics. It empirically tests the theoretically grounded hypotheses (Hypothesis 1–Hypothesis 5), thereby linking theoretical assumptions with practical findings. It contributes to the under-researched field of occupational well-being in psychiatric-psychosomatic health care settings. Also, the research provides valuable significance for the practice in designing an evidence-based intervention that incorporates the perspectives of those directly affected by these challenges.

The project is characterized by innovative aspects: the first innovative aspect lies in the provision of empirical evidence for designing organizational and individual-level interventions, taking interactions between individuals and their social environment into account. Second, it offers actionable insights for clinic administrators and policymakers. Another important outcome is that it supports sustainable workforce strategies in mental health care. Thus, the project can encourage interprofessional cooperation and mental health promotion among staff in times of increasing complexity and dynamic changes over time and in interaction with medical internet research.

The research, with its 2 phases, provides a deeper understanding of the psychological factors affecting health care professionals in the mental health sector. Beyond that, it addresses the need to improve support for psychiatric-psychosomatic care in times of digitalization and increased medical internet use.

### Conclusions

This research examines the work-related challenges faced by health care professionals in psychiatric-psychosomatic clinics. It is expected that burnout, engagement, and psychological safety will likely emerge as central mediating and moderating variables. As the findings of phase 1 serve as a basis for the development of an intervention, this research seeks to improve the well-being of health care professionals in psychiatric-psychosomatic institutions sustainably. With that, this research acts as a role model for other approaches, especially in times of increased internet-supported activities in medical workforces.

## Abbreviations

APA: American Psychological Association
BIGSSS: Bremen International School of Social Sciences
BMBF: Bundesministerium für Bildung und Forschung
BWFGB: Behörde für Wissenschaft, Forschung, Gleichstellung und Bezirke
COPSOQ: Copenhagen Psychosocial Questionnaire
DFG: Deutsche Forschungsgemeinschaft/ German Research Foundation
GDPR: General Data Protection Regulation
HAW: Hamburg University of Applied Sciences
IT: information technology
model: job demands-resources model
RCT: randomized controlled trial
SCT: social-cognitive theory
UWES: Utrecht Work Engagement Scale
